# Proteinoid Nanocapsules as Drug Delivery System for Improving Antipsychotic Activity of Risperidone

**DOI:** 10.3390/molecules25174013

**Published:** 2020-09-02

**Authors:** Liroy Lugasi, Igor Grinberg, Rivka Sabag, Ravit Madar, Haim Einat, Shlomo Margel

**Affiliations:** 1 The Institute of Nanotechnology and Advanced Materials, Department of Chemistry, Bar-Ilan University, Ramat-Gan 5290002, Israel; liroy.lugasi@gmail.com (L.L.); grinbergigori97@yandex.com (I.G.); 2 Pre-Clinical Research Center: Faculty of Life Sciences, Bar-Ilan University, Ramat-Gan 5290002, Israel; rikisabag2001@gmail.com; 3 The Leslie and Susan Gonda Multidisciplinary Brain Research Center, Bar-Ilan University, Ramat-Gan 5290002, Israel; ravit.sa@gmail.com; 4 The School of Behavioral Sciences, Tel Aviv-Yaffo Academic College, Tel Aviv 6818211, Israel; haimh@mta.ac.il

**Keywords:** proteinoid nanoparticles, nanocapsules, self-assembly, risperidone, drug delivery

## Abstract

Risperidone (RSP) is an atypical antipsychotic drug widely used to treat schizophrenia and bipolar disorder. Nanoparticles (NPs) are being developed as in vivo targeted drug delivery systems, which cross the blood-brain barrier and improve pharmacokinetics and drug effectiveness. Here, biodegradable proteinoids were synthesized by thermal step-growth polymerization from the amino acids l-glutamic acid, l-phenylalanine and l-histidine and poly (l-lactic acid). Proteinoid NPs containing RSP were then formed by self-assembly, overcoming the insolubility of the drug in water, followed by PEGylation (poly ethylene glycol (PEG) conjugation to increase the stability of the NPs in the aqueous continuous phase. These NPs are biodegradable owing to their peptide and ester moieties. They were characterized in terms of diameter, size distribution, drug loading, and long-term storage. Behavioral studies on mice found enhanced antipsychotic activity compared to free RSP.

## 1. Introduction

**Figure 1:**
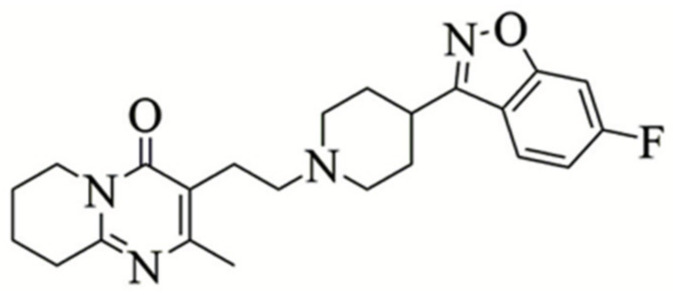
Chemical structure of risperidone.

Antipsychotics are a class of medications that are primarily used for treatment of psychoses, mainly schizophrenia and bipolar disorder [1]. Risperidone (RSP, Figure 1) is the most common atypical (second generation) antipsychotic medication, which is used to treat both positive symptoms (hallucinations, delusions, and thought disorders) and negative symptoms (emotional withdrawal, blunted effect, and loss of speech) [2].

RSP is a potent antipsychotic with antagonist binding affinity at serotoninergic, dopaminergic, adrenergic, and histaminergic receptors [3]. It is a small achiral benzoisoxazole derivative, which contains four rotatable bonds with no hydrogen bond donors and four hydrogen bond acceptors. The lipophilicity of RSP contributes to its ability to cross the blood-brain barrier (BBB), however it is susceptible to broad metabolism by hydroxylation and oxidative *N*-dealkylation to 9-hydroxyrisperidone (paliperidone), which is able to form hydrogen bonds. As a consequence, the lipophilicity decreases, and therefore also the diffusion rate through the BBB [4]. The lipophilicity also affects the metabolic activity, as lipophilic drugs tend to have greater affinity for metabolic enzymes. Hence, the higher the lipophilicity of RSP, the higher its permeability, and the faster its metabolic clearance. These opposing parameters influence the bioavailability of RSP and therefore should be taken into consideration [5]. Hydroxylation is also related to the generic polymorphism of RSP [6]. The polymorphism of the CYP2D6 gene has an important part in RSP metabolism when expressed in the blood, and demonstrates that RSP is poorly water-soluble, a weak base, and an unstable molecule. RSP is associated with several therapeutic concerns such as weight gain, dizziness, insomnia, metabolic problems, and sexual and extrapyramidal side effects [7]. This leads to inconsistent bioavailability and hampers the development of effective formulations with enhanced solubility, stability, drug efficiency and reduced side effects.

Biodegradable NPs are widely used in the field of nanomedicine owing to their many positive characteristics such as improved targeted drug delivery, biocompatibility, bioavailability and permeability, reduced toxicity, and safety [8]. Previous studies demonstrated that such NPs pose significant advantages [9] and can be used in various biomedical applications such as therapeutic and diagnostic agents for cancer [10,11], gene delivery [12], neurodegenerative disorder therapies [13], and vaccines [14]. Moreover, appropriate biodegradable NPs were demonstrated to cross the BBB, increase administration, and provide drug targeting [15,16].

Proteinoids are random polymers that consist of amino acids, which are synthesized by the thermal step-growth polymerization mechanism [17]. They are biodegradable and feature natural proteins, and hence are non-immunogenic and non-toxic [18,19,20]. Formation of ester bonds by incorporation of poly (l-lactic acid) (PLLA) during polymerization improves biodegradability and increases rigidity [21]. The major advantage of proteinoids as synthetic polymers in biomedicine is their wide spectrum, which enables tailoring the proteinoid to the specific application [22,23,24].

In an aqueous solution, proteinoids can fold to form hollow particles via self-assembly, wherein the core is composed of hydrophobic residues, e.g., phenyl rings, and the shell comprises hydrophilic residues such as carboxylates and amines. The hollow interior of the particles can be exploited to encapsulate a variety of different hydrophobic molecules, while the NP surface, comprised of chemically active groups, can be utilized to conjugate bioactive agents. Therefore, proteinoid NPs have the potential to deliver drugs to specific target sites [25].

Recent studies showed several potential psychiatric drug delivery systems using poly (lactic-*co*-glycolic acid) (PLGA) [26], a polyelectrolyte complexation between dextran-sulfate and dextran-chitosan [27] and solid lipids such as glyceryl monostearate [28]. Unlike these systems, which may possess disadvantages such as polymer growth, polydisperse distributions, bio-changes, toxicity, side effects, and high cost, the proteinoid nanocarriers presented in this study are biodegradable, non-toxic, and non-immunogenic. Moreover, RSP is encapsulated by an easy and efficient procedure, which allows enhancing and optimizing the antipsychotic effect.

Here, the acidic proteinoid poly (L-glutamic acid-L-phenylalanine-L-histidine-poly (l-lactic acid))—P(EFH-PLLA)—was designed and characterized in terms of its ability to encapsulate RSP, according to the hypothesis that nano-carrier delivery systems can stabilize the bioavailability of the drug, as well as increase its stability and solubility, thereby significantly improving its potency. The objective was to evaluate the effect of RSP-loaded proteinoid NPs in an in vivo behavioral assay relevant to psychosis.

## 2. Results and Discussion

### 2.1. Synthesis of Hollow and RSP-Loaded NPs

The synthesis of the P(EFH-PLLA) polymer was carried out as described in the methods section. The yield of the polymer was 92% (4.6 out of 5 g total monomers). The residual 8% consisted of water-insoluble proteinoid, probably due to cross-linking. The water-soluble proteinoid was then used to prepare the nanocapsules via self-assembly. The yield of this process for both hollow and RSP-loaded NPs was 100%.

### 2.2. Gel Permeation Chromatography

The P(EFH-PLLA) proteinoid was characterized in terms of molecular weight (Mw), molecular mass at the peak (Mp), number average molecular weight (Mn), and polydispersity index (PDI) given by Mw/Mn using gel permeation chromatography (GPC). The molecular weight was 142 kDa with a peak mass of 135 kDa, number average molecular weight of 139 kDa, and PDI of 1.02. P(EFH-PLLA) exhibits a high molecular weight and narrow PDI, which is unlikely to be obtained by step-growth polymerization, which is a random process, hence it resembles natural proteins [29,30].

### 2.3. ^1^H and ^13^C NMR Spectroscopy

**Figure 2:**
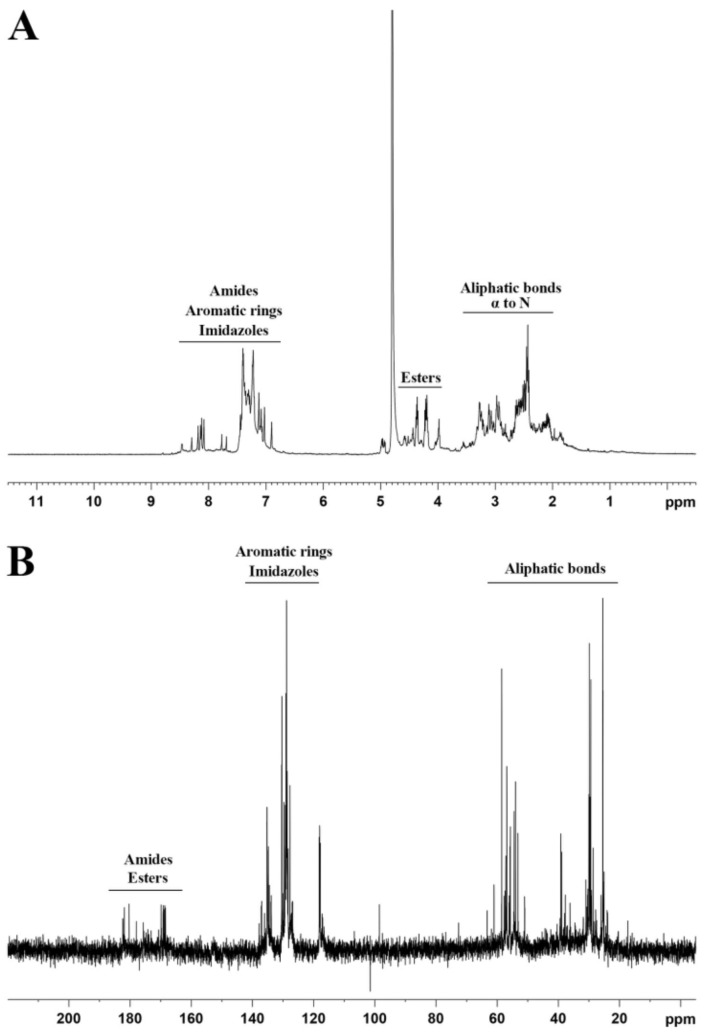
Proton (**A**) and carbon (**B**) NMR spectra of as-obtained P(EFH-PLLA) proteinoid.

Nuclear magnetic resonance (NMR) spectroscopy was applied to confirm the formation of the proteinoid polymer. Figure 2 presents ^1^H and ^13^C spectra of the as-obtained P(EFH-PLLA) proteinoid. Unlike structures of individual amino acids that present clear peaks for each group, the spectra showed multiple peak regions, indicating a polymer structure [31,32]. Protein NMR (Figure 2A) displays three main peak ranges—~2–3 ppm associated with the aliphatic bonds and α-hydrogen on the amino acids and PLLA, ~4–5 ppm, corresponding to the ester bonds between the amino acids and PLLA, and ~7–8 ppm, confirming the presence of the imidazole of histidine and the aromatic ring of phenylalanine. The ^13^C NMR spectrum (Figure 2B) also exhibits three peak regions—at ~20–60, ~120–140, and ~160–180 ppm. The first region corresponds to the aliphatic bonds on the amino acids and PLLA, the second region is in accord with the aromatic ring of phenylalanine and the imidazole of histidine, and the third region may be attributed to the amide and ester bonds that form by condensations of amino acids to each other and to PLLA, respectively. According to the spectra, it can be concluded that the thermal step-growth polymerization of the amino acids yielded a polymer-like proteinoid structure.

### 2.4. ζ-Potential Measurements

The ζ-potentials of the aqueous proteinoid NP dispersions were measured at a pH of 7.4, as described in the methods section, yielding −10 ± 4 and −16 ± 1 mV for P(EFH-PLLA) and P(EFH-PLLA)/RSP, respectively. The observed reduction corresponds well with the loading of RSP.

### 2.5. Diameter and Size Distribution

**Figure 3:**
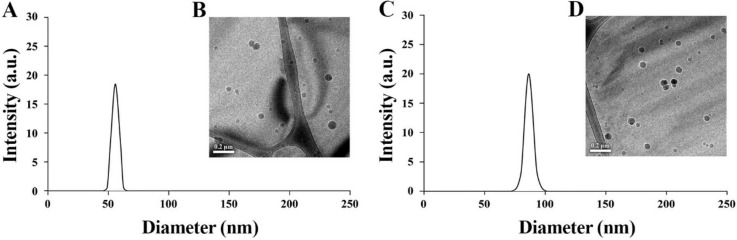
Hydrodynamic size histograms obtained by dynamic light scattering and cryogenic transmission electron microscope images of P(EFH-PLLA) (**A**,**B**) and P(EFH-PLLA)/RSP (**C**,**D**).

The diameter and size distribution of P(EFH-PLLA) and P(EFH-PLLA)/RSP were measured from FEI Tecnai G2 (Hillsboro, Oregon, USA) cryogenic transmission electron microscope (cryo-TEM) images (Figure 3). The diameter increased significantly upon drug loading (DL), from 58 ± 2 to 86 ± 3 nm, indicating successful encapsulation of RSP within the NP core. Dynamic light scattering (DLS) histograms yielded uniform populations of spherical NPs with very similar hydrodynamic diameters of 57 ± 4 and 87 ± 7 nm.

### 2.6. Drug Loading (DL) Analysis

The loading capacity of RSP drug within P(EFH-PLLA)/RSP was determined by high-performance liquid chromatography (HPLC). The initial concentration of RSP was 20% *w/w* with respect to the proteinoid. The DL (*w/w*%) of P(EFH-PLLA)/RSP after analysis was 20 ± 0.1, indicating complete encapsulation of RSP within the nanocapsules during self-assembly.

### 2.7. Cell Viability Test (XTT)

**Figure 4:**
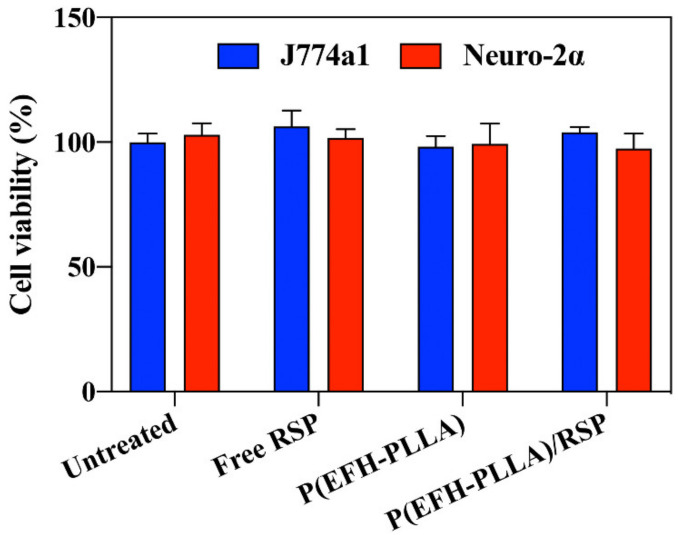
XTT cell viability assay on murine J774A.1 and Neuro-2α cells after 48 h.

In vitro cell toxicity was determined by the XTT assay on two murine cell lines. J774A.1 cells treated with 0.5 mg/mL of hollow and RSP-encapsulating P(EFH-PLLA) NPs exhibited 98 ± 4% and 104 ± 2% viability, respectively, after 48 h. Upon similar treatment, Neuro-2α cells showed similarly high viability levels of 99 ± 8% and 97 ± 7% for hollow and loaded NPs, respectively (Figure 4). As expected, similar results were observed for lower concentrations (0.1, 0.2, and 0.3 mg/mL) of NPs. These results demonstrate that the proteinoid NPs exhibit essentially no cytotoxicity compared to both control groups (cells treated with free RSP and untreated cells).

### 2.8. In Vitro Drug Release Study

An in vitro drug release study was performed at 37 °C in two media—phosphate buffered saline (PBS) and human serum. To circumvent issues of solubility and diffusion, the amount of released drug was determined indirectly by HPLC after twofold dilution in ethanol and sonication, which disrupted the nanocapsules and completely dissolved the encapsulated drug.

**Figure 5:**
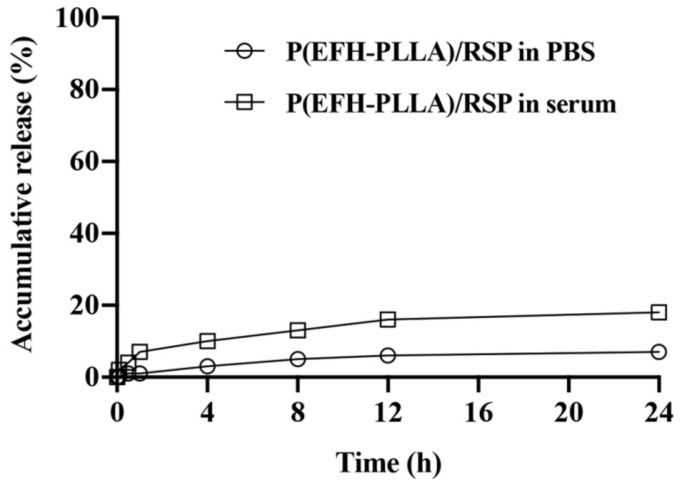
Release of risperidone (RSP) from PEGylated P(EFH-PLLA)/RSP nanoparticles (NPs) in a phosphate buffered saline (PBS) and human serum at 37 °C over a 24 h period. Each data point represents the mean of three samples.

Figure 5 shows the release profile of RSP from poly (ethylene glycol) (PEG)-conjugated (PEGylated) proteinoid NPs during a 24 h period. The release of RSP from proteinoid NPs dispersed in serum was higher compared to PBS at each time point. The maximum release in PBS, which does not contain active enzymes that can break down the ester and peptide bonds of the proteinoid NPs, was 7%, compared to 18% in human serum, which contains a high concentration of active enzymes that target the ester and peptide bonds. As the proteinoid differs from natural proteins in secondary and tertiary structure, cleavage of the bonds requires a longer amount of time, and drug release is slower.

### 2.9. Biodistribution Study

**Figure 6:**
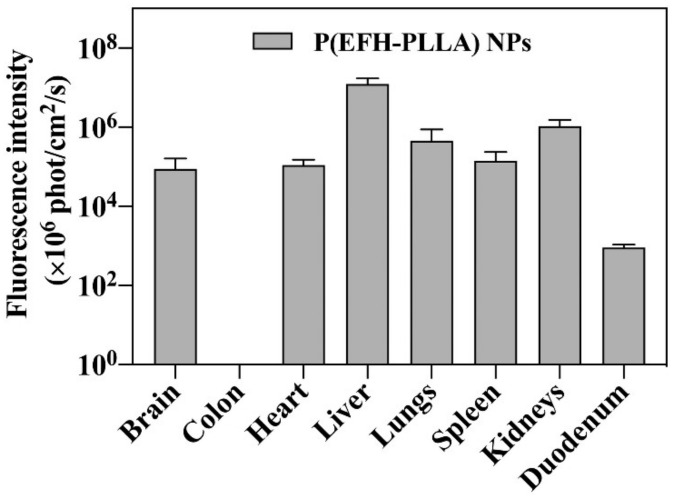
Biodistribution of P(EFH-PLLA) NPs. One hundred µL of Cy7-conjugated NPs (0.2 mg/mL) were injected IV via the tail vein. Mice were sacrificed 4 h post injection, and organs were harvested.

The in vivo biodistribution of Cy7-conjugated PEGylated P(EFH-PLLA) NPs was investigated in mice as shown in Figure 6. Fluorescence was exhibited in all harvested organs with the exception of the colon, probably due to fast clearance in this organ. The significant fluorescence in the brain (~1%) indicates that the NPs are capable, to some extent, of passing through the BBB.

### 2.10. Long-Term Storage Stability

When NPs are stored for long periods of time in aqueous media, physical instability frequently occurs, which restricts their use. Therefore, aqueous dispersions of NPs are routinely stored in the presence of an appropriate cryoprotectant. Common efficient cryoprotectants include trehalose, mannitol, sorbitol, and glycerol [33]. The hollow and drug-containing proteinoid dispersions were freeze-dried in the presence of 1% (*w*/*v*) trehalose, a natural alpha-linked disaccharide [34].

Notably, in the absence of trehalose, RSP-encapsulating NPs aggregated and could not be redispersed, while in the presence of trehalose, redispersion in an aqueous continuous phase did not affect the particle size or the DL. Furthermore, the dried powder can be kept for at least one year.

### 2.11. Behavioral Studies

Psychostimulant-induced behaviors are frequently used to model facets of schizophrenia and mania, especially in the context of screening potential novel treatments. A number of psychostimulants are used in this context, however the more established drug for this model is amphetamine [35]. In the amphetamine-induced hyperactivity model, single or multiple doses of amphetamine administered to a model animal induce hyperactivity—including increased distance and speed—as well as other changes in behavior. This constitutes an accepted screening model for antipsychotic effects [36] as antipsychotics and other mood stabilizers were repeatedly demonstrated to ameliorate amphetamine-induced behavioral changes.

The effects of RSP encapsulated in a nano-carrier delivery system were examined using the amphetamine-induced screening model. The results clearly indicate that P(EFH-PLLA)/RSP NPs have a strong and significant effect on all measured parameters including distance, speed, and time in the center. Moreover, in most cases, the effect was significantly larger compared with similar doses of free RSP, confirming the expected advantage of administration using a nano-carrier delivery system.

#### 2.11.1. Distance and Speed

##### Distance

**Figure 7:**
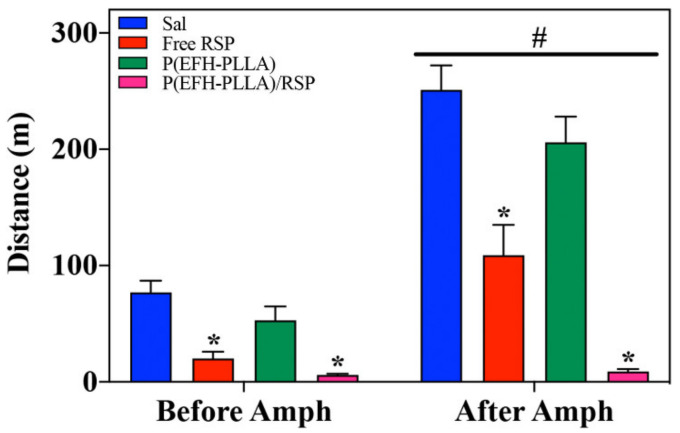
Distance analysis using ANOVA (analysis of variance) of open field test with hollow and RSP-loaded NP treatment 0–30 min prior to amphetamine administration and 30–60 min post amphetamine uptake. Asterisks (*) denote statistical significance versus saline and the hashtag (#) signifies the effect of amphetamine administration.

As expected, amphetamine administration resulted in an increase in the distance travelled in the arena [F(1,28) = 171.9, *p* < 0.001] as well as in a significant effect of treatment [F(3,28) = 28.2, *p* < 0.001] and interactions [F(3,28) = 23.0, *p* < 0.001]. RSP administered before amphetamine reduced activity [F(3,28) = 15.3, *p* < 0.0001; post-hoc: Free RSP ≠ Sal (*p* < 0.0001); P(EFH-PLLA)/RSP ≠ Sal (*p* < 0.0001)]. Effect size (Cohen’s d) versus saline for free RSP and P(EFH-PLLA)/RSP was 2.46 and 3.57, respectively. RSP also reduced amphetamine-induced hyperactivity [F(3,28) = 28.7, *p* < 0.0001; post-hoc: Free RSP ≠ Sal (*p* < 0.001); P(EFH-PLLA)/RSP ≠ Sal (*p* < 0.0001); free RSP ≠ P(EFH-PLLA)/RSP, *p* = 0.016]. Effect size (Cohen’s d) was 2.12 for free RSP versus saline, 5.77 for P(EFH-PLLA)/RSP versus saline, and 1.9 for free RSP versus P(EFH-PLLA)/RSP (Figure 7).

##### Speed

**Figure 8:**
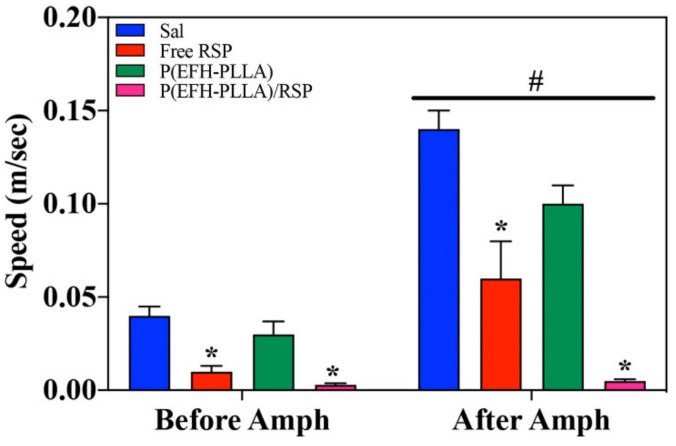
Mean speed analysis using ANOVA of open field test with hollow and drug-loaded NPs treated 0–30 min prior to amphetamine administration and 30–60 min post amphetamine uptake. Asterisks (*) denote statistical significance versus saline and the hashtag (#) signifies the effect of amphetamine administration.

As expected, amphetamine administration resulted in increased speed [F(1,28) = 172.2, *p* < 0.001] as well as in a significant effect of treatment [F(3,28) = 28.1, *p* < 0.001] and interactions [F(3,28) = 23.0, *p* < 0.001]. RSP administered before amphetamine reduced speed [F(3,28) = 15.2, *p* < 0.0001; post-hoc: Free RSP ≠ Sal (*p* = 0.001); P(EFH-PLLA)/RSP ≠ Sal (*p* < 0.0001)]. There was also a trend in the difference between P(EFH-PLLA)/RSP and free RSP (*p* = 0.055). Effect size (Cohen’s d) for free RSP versus saline was 1.69 and for P(EFH-PLLA)/RSP versus saline 1.74. RSP reduced amphetamine-induced speed as well [F(3,28) = 28.7, *p* < 0.0001; post-hoc: Free RSP ≠ Sal (*p* = 0.001); P(EFH-PLLA)/RSP ≠ Sal (*p* < 0.0001); free RSP ≠ P(EFH-PLLA)/RSP, *p* = 0.018]. Effect size (Cohen’s d) was 1.69 for free RSP versus saline, 6.33 for P(EFH-PLLA)/RSP versus saline, and 1.29 for free RSP versus P(EFH-PLLA)/RSP (Figure 8).

Both free and encapsulated RSP significantly reduced locomotion. Interestingly, RSP also reduced locomotion prior to amphetamine administration. This is a common effect when using antipsychotic drugs, which are known to reduce activity in intact animals [37]. The enhancement due to encapsulation is clearly seen in the amphetamine-treated mice where administration of P(EFH-PLLA)/RSP had a significantly stronger effect than free RSP (Figure 7 and Figure 8).

The difference between administration before and after amphetamine uptake is not only statistically significant but is also associated with effect magnitudes above 1.2 that are considered “very large” [38]. However, it is important to note that locomotor activity in mice that received encapsulated RSP was reduced to near zero including minutes of freezing (data not shown). This suggests that the administered dose was in the high range, similar to doses that induce catatonia in animals or humans. However, this has no bearings on the current research question of whether the encapsulated drug has behavioral effects and whether these effects are comparable to or better than those of free RSP. Clearly, a more detailed dose-response study will help identify the appropriate dosage for use in further investigations with animal models and eventually in patients.

#### 2.11.2. Time in Center

Time in center is considered to be related to anxiety and serves as one of the standard measurements for anxiety-like behavior in rodents [39]. Amphetamine administration resulted in less time spent in the center of the arena [F(1,28) = 6.48, *p* = 0.017] as well as in a significant effect of treatment [F(3,28) = 15.43, *p* < 0.001]. Treatment before amphetamine administration significantly increased the center time [F(3,28) = 13.6, *p* < 0.0001; post-hoc: P(EFH-PLLA)/RSP ≠ all other groups (*p* < 0.001)]. Effect size (Cohen’s d) was 0.24 for free RSP versus saline, 2.46 for P(EFH-PLLA)/RSP versus saline, and 1.7 for free RSP versus P(EFH-PLLA)/RSP.

**Figure 9:**
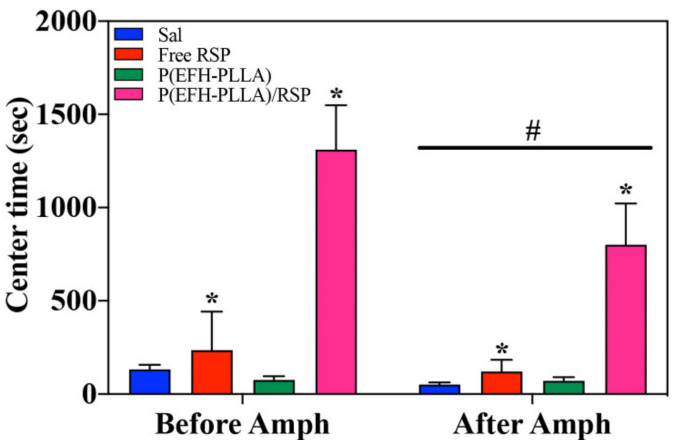
Center time analysis using ANOVA of open field test with hollow and drug-loaded NP treatment 0–30 min prior to amphetamine administration and 30–60 post amphetamine uptake. Asterisks (*) denote statistical significance versus saline and the hashtag (#) signifies the effect of amphetamine administration.

Treatment also antagonized amphetamine-induced reduction of center time [F(3,28) = 9.67, *p* < 0.001; post-hoc: P(EFH-PLLA)/RSP ≠ all other groups (*p* < 0.005)]. Effect size (Cohen’s d) was 0.55 for free RSP versus saline, 1.68 for P(EFH-PLLA)/RSP versus saline, and 1.47 for P(EFH-PLLA)/RSP versus free RSP (Figure 9).

Effects were similar to the locomotor activity. Amphetamine reduced the time spent in the center of the arena, while RSP ameliorated this reduction before and after amphetamine administration. However, in both cases, in contrast with the locomotion measurements, only encapsulated RSP was shown to significantly increase the center time.

Amphetamine was previously reported to have an anxiogenic effect in this type of model, resulting in a reduction of center time in both rats and mice [40]. In this context, it is tempting to suggest that encapsulated RSP has an anxiolytic effect. However, the data is not strong enough to fully support this notion. Alternatively, it is possible that the increased time spent in the center of the arena is a consequence of the large reduction in locomotion that included freezing during parts of the session. Clearly, additional tests for anxiety-like behavior that are less dependent on exploratory behavior and activity levels are needed to resolve this issue.

## 3. Materials and Methods

### 3.1. Materials

The following analytical-grade chemicals were purchased from commercial sources and used without further purification: l-glutamic acid (E), l-phenylalanine (F), l-histidine (H), sodium hydroxide (NaOH), super-pure HPLC water, acetonitrile and trifluoroacetic acid, sodium chloride (NaCl), dimethyl sulfoxide (DMSO), deuterium oxide (D_2_O) NMR solvent, Cyanine7-NHS (Cy7-NHS), and trehalose were all purchased in ≥98% purity from Sigma (Rehovot, Israel). Poly (l-lactic acid) (PLLA) MW 2 kDa was purchased from Polysciences (Warrington, PA, USA), methoxy polyethylene glycol succinimidyl succinimide (M-PEG-NHS, MW 5 kDa) was obtained from JenKem Technology (Plano, TX, USA), and risperidone was obtained from Teva Pharmaceutical Industries Ltd. (Kfar Saba, Israel). Water was purified by passing deionized water through an Elgastat Spectrum reverse osmosis system from Elga Ltd. (High Wycombe, UK). Dialysis membranes (1000 Da), Dulbecco’s modified Eagle’s medium (DMEM), PBS, glutamine, penicillin/streptomycin, and cell proliferation kit (XTT) were purchased from Biological Industries (Bet Haemek, Israel). Neuro-2α and J774A.1 murine macrophage cell lines were purchased from ATCC (Manassas, VA, USA). Male ICR (CD-1^®^) eight-week-old mice were purchased from Harlan Biotech (Rehovot, Israel).

### 3.2. Methods

#### 3.2.1. Synthesis of P(EFH-PLLA) and P(EFH-PLLA)/RSP NPs

The proteinoid polymer poly(EFH-PLLA), briefly P(EFH-PLLA), was synthesized with equal amounts of amino acids and 10% (*w*/*w*) of 2 kDa PLLA monomer, as described in the literature [41]. RSP-loaded proteinoid NPs were prepared by self-assembly.

Briefly, 100 mg of P(EFH-PLLA) powder was added to 10 mL of a 10^−5^ M aqueous NaCl solution, heated to 80 °C and stirred at 250 rpm for 30 min. RSP powder 20 weight % relative to the proteinoid was dissolved in DMSO (0.2 mL) and heated to 80 °C. The RSP solution was gradually added to the heated P(EFH-PLLA) solution. The obtained homogeneous solution was then left to cool slowly to room temperature to form P(EFH-PLLA)/RSP NPs. This was followed by extensive dialysis using a cellulose dialysis membrane with a molecular weight cut-off (MWCO) of 1000 Da against distilled water to remove the DMSO. The obtained proteinoid NP aqueous dispersion was filtered through a 3 µm glass microfiber membrane syringe filter to remove the untrapped RSP.

The NPs were then PEGylated with M-PEG-NHS, Mw 5 kDa. Briefly, 100 µL of M-PEG-NHS dissolved in PBS (50 mg/mL) was added to P(EFH-PLLA)/RSP (10 mg/mL) dispersed in sodium bicarbonate (2 mM, pH = 8) and stirred at 150 rpm for 2 h at room temperature. The obtained PEGylated P(EFH-PLLA)/RSP aqueous dispersion was dialyzed through a cellulose membrane (1000 Da MWCO) against super-purified water to remove excess material. Hollow P(EFH-PLLA) NPs without RSP were prepared in the same manner. The yield was determined by freeze-drying. Briefly, the aqueous NP dispersion was freeze-dried with liquid nitrogen under vacuum to form a powder, which was then weighed.

#### 3.2.2. Molecular Weight Analysis

The molecular weights and polydispersity indices of the dried P(EFH-PLLA) proteinoid were determined at 25 °C using GPC, consisting of a Waters Spectra Series P100 isocratic HPLC pump with an ERMA ERC-7510 refractive index detector and a Rheodyne (Cotati, CA, USA) injection valve with a 20 µL loop (Waters, MA, USA). The sample was eluted with super-pure HPLC water through a linear BioSep SEC-s3000 column (Phenomenex, Torrance, CA, USA) at a flow rate of 1 mL/min. The molecular weight of the proteinoid was determined relative to PEG standards (Polymer Standards Service-USA, Silver Spring, MD) with a molecular weight range of 100–450,000 Da, human serum albumin (HSA, 67 kDa), and bovine plasma fibrinogen (340 kDa) using the Clarity chromatography software version 2.7.3.498 (DataApex, Prague, Czech Republic).

#### 3.2.3. NMR Spectroscopy of P(EFH-PLLA) Proteinoid

NMR spectroscopy was carried out using an Avance I 400 MHz spectrometer (Bruker, Rheinstetten, Germany). Briefly, 40 mg of P(EFH-PLLA) were dissolved in 1 mL of D_2_O, and the solution was subjected to ^1^H and ^13^C NMR spectroscopy at 400.1 and 100.6 MHz, respectively.

#### 3.2.4. ζ-Potential Measurements of P(EFH-PLLA) and P(EFH-PLLA)/RSP NPs

The proteinoid NP surface potentials were measured in aqueous dispersion at pH of 7.4 at a concentration of 10 mg/mL using a Zetasizer 3000 ζ-potential analyzer (HSa model, Malvern Instruments Company, Malvern, UK).

#### 3.2.5. Diameter and Size Distribution

The diameter and size distribution of the aqueous NP dispersions were measured by cryo-TEM. Briefly, a small droplet was placed on a perforated lacey carbon film supported on a TEM copper grid. The drop was blotted with a piece of filter paper, resulting in formation of a thin film of 100–300 nm. The specimen was subsequently plunged into a reservoir of liquid ethane cooled by liquid nitrogen to ensure vitrification (rapid freezing) and prevent ice crystal formation. The vitrified specimen was transferred under liquid nitrogen and mounted on a cryogenic sample holder cooled to −170 °C. All samples were observed under low-dose conditions. Vitrified samples were examined using a FEI T12 G2 Cryo-TEM (Hillsboro, Oregon, USA) operating at 120 kV and equipped with a Gatan 626 cryo-holder system. The mean diameter was determined by measuring at least 200 particles using the image analysis software AnalySIS Auto version 3.2 (Soft Imaging System GmbH, Münster, Germany). In addition, the hydrodynamic diameter and size distribution of the aqueous NP dispersions were measured at room temperature with a particle DLS analyzer (Nanophox, Sympatec GmbH, Clausthal-Zellerfeld, Germany).

#### 3.2.6. HPLC RSP DL Analysis

HPLC analysis of RSP DL was carried out using a Spectra System HPLC equipped with a UV/vis detector (Thermo Scientific, Waltham, MA, USA) and a reverse phase C18 column (75 × 4.6 mm, Phenomenex). The mobile phase was water and acetonitrile, both containing 0.1% aqueous solution of TFA at a flow rate of 1 mL/min at 25 °C, and the wavelength was set at 285 nm [42]. Calibration standard solutions with 60–200 µM of RSP were prepared by diluting an appropriate volume of stock solution in ethanol.

RSP-loaded NPs were diluted two-fold with ethanol and sonicated in an ice-water bath for 20 min to disrupt the nanocapsules. The sonication and added ethanol caused the proteinoid NPs to disassemble and elute the RSP. The injection volume was set to 10 µL for all standard samples in the range of 60–200 µM. The weight of drug in each sample was calculated using the calibration curve.

#### 3.2.7. Cell Viability Test (XTT)

In vitro toxicity was assessed using an XTT (2,3-bis-(2-methoxy-4-nitro-5-sulfophenyl)-2H-tetrazolium-5-carboxanilide salt) assay with murine macrophage J774A.1 and Neuro-2α cell lines. Cells were seeded in a 96-well plate at a density of 1 × 10^4^ cells/well in 100 µL culture medium and grown in a humidified 5% CO_2_ atmosphere at 37 °C. The NPs were freshly dispersed in water containing 1% DMSO aqueous solution and added to a 95% confluent cell culture in culture medium. Cells were treated with 10 µg/mL of RSP or with P(EFH-PLLA) or P(EFH-PLLA)/RSP NPs at a concentration of 0.1, 0.2, 0.3, or 0.5 mg/mL.

Free RSP and untreated cells were used as controls. Cell cultures were further incubated at 37 °C in a humidified 5% CO_2_ incubator, and cell viability was analyzed after 48 h, as suggested in the XTT protocol. The experiment was repeated twice. All samples were tested in sixfold and analyzed by UV spectrophotometer at 492 nm with reference wavelength of 620 nm.

#### 3.2.8. In Vitro Drug Release Study

In vitro release of RSP from PEGylated RSP-loaded NPs was studied. Dry samples were suspended in PBS at a final concentration of 1 mg/mL. Then, 0.4 mL of PBS dispersion were added to 1.6 mL of either PBS or human serum. All samples were placed in a shaker with constant agitation at 37 °C for 24 h and filtered through a centrifugation tube (Vivaspin 30 kDa MWCO). Supernatant samples were taken at each time interval. The concentration of the remained and trapped RSP-loaded NPs in each sample were determined by HPLC, followed by sonication to release the RSP from the NPs as described in Section 3.2.4. RSP concentrations were determined using a calibration curve of known RSP concentrations as described above. The study was performed in triplicate.

#### 3.2.9. Biodistribution of P(EFH-PLLA) NPs

For the biodistribution study, the near IR (NIR) dye Cyanine7 NHS ester (Cy7-NHS) was conjugated to residual amine groups on the surface of PEGylated hollow P(EFH-PLLA) NPs. Briefly, after coupling of M-PEG-NHS to the NPs, 50 µL of a Cy7-NHS solution in DMSO (10 mg/mL) was added to the NP aqueous dispersion and stirred at 150 rpm for an additional 2 h at room temperature. The NIR fluorescent conjugated NP dispersion was then extensively dialyzed through a cellulose membrane (1000 Da MWCO) against distilled water to remove the DMSO and excess Cy7.

The biodistribution of the Cy7 fluorescent P(EFH-PLLA) NPs was studied on normal eight-week-old male BALB/C mice weighing 25–30 g at the time of the experiment (repeated twice with five NP treated mice and one nontreated mouse as a negative control). For this purpose, 100 µL of the Cy7-conjugated NPs and PBS dispersion (0.01 mg/kg body weight) were administered to the mice via IV injection to the tail vein at a concentration of 0.3 mg/kg. Blood samples were taken immediately post injection at 0, 30 min, 1 and 4 h. The mice were euthanized by CO_2_ inhalation after 4 h of the experiment, and blood and organs (brain, colon, heart, lungs, liver, spleen, kidneys, and duodenum) were harvested for imaging.

Fluorescence images were acquired using a Maestro II in vivo fluorescence imaging system (Cambridge Research and Instrumentation Inc., Woburn, MA) equipped with a fiber-delivered 300 W xenon excitation lamp, and images were acquired from λ = 500–950 nm by a 1.3 megapixel CCD camera (Sony Inc., ICX285 CCD chip, Tokyo, Japan). A deep red excitation/emission filter set was used for our experiments (λex: 700–770 nm, λem > 780 nm). The liquid crystal tunable filter (LCTF) was programmed to acquire image cubes from λ = 780 to 840 nm with an increment of 10 nm per image. The camera was set to exposure times of 20,000 ms (brain, spleen), 5000 ms (blood), 3000 ms (liver, kidneys, duodenum), 2000 ms (heart), 1000 ms (lungs), and 400 ms (colon). Fluorescence intensity measurements were performed using the ImageJ NIH (National Institutes of Health) software version 1.48v.

#### 3.2.10. Long-Term Storage Stability Study

The long-term storage of hollow and drug-loaded P(EFH-PLLA) NPs was investigated using freeze-drying. Briefly, 10 mg of trehalose were added to 1 mL of NP aqueous dispersion (10 mg/mL), followed by lyophilization and storage at 4 °C. NP powders were redispersed in double-distilled water to their original concentration (1 mL) and recharacterized (diameter and size distribution) by cryo-TEM, and the DL was analyzed by HPLC. The dried powders were redispersed once a month over a one year period.

#### 3.2.11. Behavioral Studies

##### Animals

Male ICR (CD-1^®^) eight-week-old mice weighing 25–30 g at the start of the experiment were purchased from Harlan Biotech and maintained four per cage in an animal facility with standard conditions (12 h/12 h light/dark cycle, 20 ± 1 °C and ad lib access to standard rodent chow and water). All experiments complied with the ARRIVE guidelines (Animal Research: Reporting of In Vivo Experiments) and were carried out in accordance with the Israeli Ministry of Health and NIH Guides for the Care and Use of Laboratory Animals (NIH Publications No. 80-23). All procedures were approved by the Bar-Ilan University Institutional Animal Care and Use Committee (IACUC, protocol #16-09-2019).

##### Treatment Protocol

Mice were treated with 100 µL of saline (control), free RSP (10 mg RSP dissolved in 1% *v/v* DMSO and 5 mL of super-purified water), or hollow or RSP-loaded NP dispersion (at a final concentration of 2.0 mg/mL) injected IV. Free RSP was dissolved in 10 mL of a 1% DMSO aqueous solution to a 0.3 mg/kg concentration and 100 µL injection volume. The solution was injected IV via the tail vein. All injections were administered 30 min prior to testing.

##### Testing

Animals underwent the amphetamine-induced behavior test, which is a standard screening test for antipsychotic-like effects in rodents, also performed with RSP [43]. Thirty minutes after the last RSP/control drug administration, the mice were transferred to a dedicated room and placed in an open field arena (40 × 40 cm) with 30 cm walls made of black Plexiglas where their behavior was followed using a video-tracking system (Any-Maze, Stoelting, IL, USA). After 30 min in the open field, the mice were briefly taken out and injected IP with amphetamine dissolved in saline at a 5.0 mg/kg dose and 10 mL/kg volume, after which they were immediately returned to the open field arena for an additional 30 min. This procedure allowed an enclosed subject comparison (before and after amphetamine administration) common to similar studies [44]. At the end of the session, the mice were placed back in their home cages and returned to the colony room. The open field apparatus was cleaned with a 10% alcohol solution before the next session.

##### Statistical Analysis

Data for distance travelled, locomotion speed, and time in the center area of the open field was extracted from the video-tracking software and analyzed using a mixed analysis of variance (ANOVA) with NP injection treatment as the main factor and amphetamine treatment as a repeated measures factor (before and after amphetamine injection). Significant effects were followed by one-way ANOVA for the different phases (before and after amphetamine administration) and Scheffe post-hoc tests. Effect sizes (Cohen’s d) were calculated using an online calculator (https://www.uccs.edu/lbecker). One mouse from group Prot.1/RSP was excluded from the statistical analysis as an outlier (more than two standard deviations from the group mean).

## 4. Conclusions

In this study, a new drug delivery system was designed and investigated for the antipsychotic drug RSP. For this purpose, a proteinoid was synthesized in high yield from L-glutamic acid, phenylalanine, histidine and PLLA by step-growth polymerization. The P(EFH-PLLA) proteinoid was characterized by GPC, yielding a high molecular weight of 142 kDa with narrow PDI of 1.02, similar to natural polymers. The polymerization and polymer structure were confirmed by ^1^H and ^13^C NMR spectroscopy.

The proteinoid was self-assembled with very high efficiency in an aqueous solution to form hollow and RSP-loaded P(EFH-PLLA) NPs, followed by PEGylation. The stability of the NPs was assessed by measuring the ζ-potential, and they were characterized in terms of diameter and size distribution by cryo-TEM, which yielded nano-sized diameters of 58 ± 2 and 86 ± 3 nm, respectively. Very similar hydrodynamic diameters were found by DLS.

The DL was analyzed using HPLC, which confirmed that RSP was completely encapsulated within the P(EFH-PLLA) NPs. An in vitro cytotoxicity study demonstrated that both NPs are non-toxic, while an in vivo biodistribution study found significant fluorescence in mouse brain, which supports the hypothesis that proteinoid NPs can cross the BBB and successfully deliver the encapsulated drug.

Long-term storage was successfully achieved by the addition of trehalose to the NP aqueous dispersion. The similar diameter and size distribution before and after lyophilization confirmed this methodology for storage of NPs as a powder for long periods of time.

The RSP-loaded NPs underwent behavioral testing in mice using the amphetamine-induced hyperactivity screening model. The results of the current study clearly indicate the strong and significant behavioral effect of encapsulated RSP on activity measurements—distance, speed, and center time—in an open field, before and after amphetamine administration. Treatment with P(EFH-PLLA)/RSP NPs significantly reduced activity and speed and increased the time spent in the center of the open field. Moreover, the effect of the encapsulated drug on amphetamine-induced behavior was significantly stronger than an equal dose of free RSP.

The enhanced antipsychotic effect is in accord with slower kinetics and metabolism assumed for encapsulated versus free RSP and consequent improved drug delivery to the target site in the brain. Administration of drug-loaded proteinoid NPs could therefore offer an advantageous approach for RSP and similar antipsychotic medications, as well as for other hydrophobic drugs. Further studies are ongoing in our laboratory.
